# Host Defense against Common Early Life-Threatening Infections

**DOI:** 10.1155/2013/350808

**Published:** 2013-09-05

**Authors:** Robert Bortolussi, Philipp Henneke, Tobias Kollmann

**Affiliations:** ^1^IWK Health Centre, 5850 University Avenue, Halifax, NS, Canada B3H 1L6; ^2^Zentrum für Kinder und Jugendmedizin & Centrum für Chronische Immundefizienz (CCI), Breisacherstraße 117-2.OG, 79106 Freiburg, Germany; ^3^Department of Pediatrics, Division of Infectious Diseases, University of British Columbia, Vancouver, BC, Canada V5Z 4H4

## 1. Introduction

Neonates suffer more severely and die more often than adults from a wide range of infections [1]. Although quantitative differences between neonatal and adult immune capacity are known, the molecular basis for the transition of immunologic function from fetal to postnatal life has remained a mystery. However, over the past decade, there has been an explosion of knowledge on immunity of the newborn and its importance to early host response to infection. Recent advances in developmental immunology now allow us to better understand the mechanisms underlying the susceptibility of neonates to many bacterial and viral infections [2, 3]. In addition, neonatal intestinal microbial ecology is now appreciated to play a role in host defenses and in the pathogenesis of neonatal necrotizing enterocolitis (NEC) [4]. In this special issue, we have collected reviews and papers to provide insight in immunologic adaptation of the neonate to its new environment and the consequences of this transition to newborn host defense mechanisms to bacterial and viral infection.

## 2. Immunologic Adaptation

The neonatal intestinal tract faces a particular challenge in adapting to the complex microbial ecosystem that develops immediately after birth. Host immunity and the microbiome develop through reciprocal interactions. The review by E. Tourneur and C. Chassin shows how aberrant development of the mucocutaneous intestinal barrier contributes to inflammatory disorders, such as NEC. This paper and that of D. J. Hackam et al. highlight the specific contribution of the receptor for bacterial endotoxin, Toll-like receptor 4 (TLR4), and related signaling events in normal development and NEC pathogenesis. Intestinal epithelial cells are innate immune competent and shape the development of intestinal immunity. Bacterial toxins have also been appreciated as critical effectors of several pathogens that threaten newborn infants. Their role in invasive neonatal infections is reviewed by A. F. Sonnen and P. Henneke. Given recent advances in toxin structure and membrane action, appreciating changes in immune cell signaling in newborns will be an essential step to understand pathogenic mechanisms of neonatal disease.

## 3. Bacterial Pathogenesis in the Newborn

While barriers such as skin and mucous membranes are more readily breached in the newborn compared to the adult, the infectious risk in early life is higher irrespective of route of infection [2, 3]. The clinically most relevant reason identified for these observations is the reduced ability of the newborn to prevent systemic spread of pathogens [5]. For example, A. M. Sherrid and T. R. Kollmann review how local cell-autonomous, as well as systemic innate and adaptive immunity of the newborn fails to restrict the spread of *L. monocytogenes*. And K. V. Driessche et al. highlight that the diminished proinflamatory cytokine capacity of the newborn following infection with *M. tuberculosis* allows bacterial spread. The reviews by M. R. P. Coombs et al. for *Staphylococcus* species and E. A. Marchant et al. for coagulase-negative *Staphylococcus* emphasize that the inability for preterm and full-term newborns to localize infection leads to high morbidity and mortality early in life.

## 4. Viral Pathogenesis in the Newborn

The paper by S. Gantt and W. J. Muller reviews the immunologic basis for severe neonatal herpes disease and potential strategies for therapeutic intervention. They then explore differences between the immune system of newborns and those of older children and adults, which predispose them to severe infections. Two papers focus on another major viral pathogen, cytomegalovirus (CMV). In the first, M. Schleiss reviews the present state of knowledge on the immune responses of the fetus and newborn infant to CMV. With this as a background, the reader can better understand the rationale for the novel vaccine strategy proposed by M. Leviton et al. to prevent congenital infection, using an attenuated CMV vaccine. 

It is our hope that this series of reviews and important new information will provide the reader with a clearer understanding of newborn host defense mechanisms and potential therapeutic strategies.

*Robert Bortolussi* *Philipp Henneke* *Tobias Kollmann*
